# Thos. T. De Graffenreid

**Published:** 1877-07

**Authors:** M. E. Tarvin


### OBITUARY.

Thomas T. de Graffenreid, D. D. S., a regular graduate of the Baltimore College of Dental Surgery, of the class of 1854, died at Oakland, Colorado Co., Texas, at 11 o'clock P. M., 16th of August, 1875, of consumption, to which was added cancer of the tongue. He was a native of Mechlenburg, Virginia, but a resident of this county for twenty years. At the breaking out of the war, he enlisted in Capt. Jno. C. Upton's Co., of this county, and went to his native state to do battle for the cause he loved so well; at Richmond the company was attached to the 5th Texas Regiment, Hood's Brigade, and known as Company B. He was never very robust, but served as a private in the Company to the close of the war. Returning to Columbus, Texas, he resumed and pursued the practice of Dentistry, until a few months before his death. His failing health forced him to discontinue the practice. His afflictions were borne with patient resolution and fortitude; he was a kind-hearted husband and father, and one of our most honored, worthy, and useful citizens. He was an exemplary member of the dental profession, was sober, upright and prudent in his daily walks of life. He was frugal, but owing to the small practice this place and county afforded, he lived up to his income, and left but little to his widow and three small children. Peace to his ashes.

M. E. Tarvin, D. D. S.

